# Supplementary material for: Comparison of Intravenous Microdialysis and Standard Plasma Sampling for Monitoring of Vancomycin and Meropenem Plasma Concentrations—An Experimental Porcine Study
Source: Antibiotics (Basel). 2023 Apr 21;12(4):791. doi: 10.3390/antibiotics12040791 (PMC10135263; doi:10.3390/antibiotics12040791)
Supplement: Supplementary file 1 [file antibiotics-12-00791-s001.zip › antibiotics-2271482-supplementary.pdf]

File S1. The principle of a clinical standard homogeneous enzyme immunoassay method for vancomycin.

Test principle: Homogeneous enzyme immunoassay technique (EMIT)

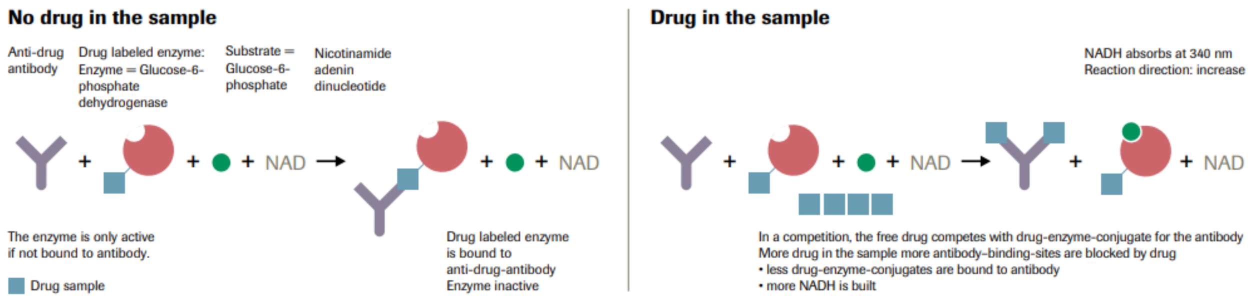

- The assay is based on competition between drug in the sample and drug labeled with the enzyme glucose-6-phosphate dehydrogenase (G6PDH) for antibody binding sites
- Enyzme activity decreases upon binding to the antibody, so the drug concentration in the sample can be measured in terms of enzyme activity
- Active enzyme converts oxidized NAD to NADH, resulting in an absorbance change that is measured spectrophotometrically
